# *Mycobacterium mucogenicum* meningitis due to external ventricular drain

**DOI:** 10.1099/acmi.0.000167

**Published:** 2020-09-03

**Authors:** Salwa Moiz, Omar Rahman, Mark Morcos, Asma Siddiqui, Usman Bin Hameed

**Affiliations:** ^1^​ Center for Aging Research, Regenstrief Institute, Indianapolis, USA; ^2^​ Department of Pulmonary and Critical Care, Indiana University School of Medicine, Indianapolis, USA; ^3^​ The Aga Khan University School of Medicine, Karachi, Pakistan

**Keywords:** external ventricular drain, meningitis, non-tuberculous *Mycobacterium*

## Abstract

**Introduction:**

*
Mycobacterium mucogenicum* is a rare non-tuberculous organism associated with catheter-related infections when pathogenic in humans. We present the first case of an external ventricular drain (EVD)-associated *M. mucogenicum* meningitis.

**Case presentation:**

A 55-year-old woman had EVD placement for obstructive hydrocephalus following traumatic subarachnoid haemorrhage. Cerebrospinal fluid (CSF) was obtained 5 days later for fever and neurological changes. *M. mucogenicum* was ultimately isolated from the CSF and the patient was placed on appropriate antibiotics. Her management included replacement of the EVD and a prolonged course of anti-mycobacterial antibiotics. CSF findings showed her response to therapy and neurological exam improved after 6 weeks.

**Conclusion:**

*
M. mucogenicum* infections are very rare and existing reports indicate that it may be a device- or catheter-related pathogen. This microorganism has not been previously associated with an EVD. Ours may be the first documented report of EVD-related *M. mucogenicum* infection.

## Introduction

*Mycobacterium mucogenicum *, formerly called '* Mycobacterium chelonae *-like' organism, is a type of rapidly growing non-tuberculous mycobacterium (NTM). Infections reported in humans are due to long-term central intravenous catheters and less frequently with peritoneal catheters [1–4].

*M. mucogenicum* has also been reported to be associated with central nervous system (CNS) infections, respiratory infections, skin and soft tissue infection, hepatitis, sepsis, bacteraemia, catheter sepsis, pneumonia, subcutaneous abscess, cellulitis, osteomyelitis, lymphadenitis, surgical wound infection and peritonitis [2, 5–8]. Both immunosuppressed and immunocompetent patients may develop CNS disease. There have been two reported cases of fatal meningitis in immunocompetent patients [9]. We present a case of external ventricular drain (EVD)-associated CSF infection with *M. mucogenicum *, not previously reported in the literature.

## Case report

A 55-year-old female presented with altered mental status and hydrocephalus. She had blunt head trauma associated with subarachnoid and intraventricular haemorrhage. A head computed tomography scan showed blood in occipital horns bilaterally and increased ventriculomegaly of the lateral, third and fourth ventricles. An EVD was placed at the bedside. She subsequently developed worsening altered mental status, fever and tremors with rising serum white blood cell count. Cerebrospinal fluid (CSF) was obtained from the EVD on day 5 as part of initial work up that showed pleocytosis and hypoglycorrhachia. CSF studies were repeated 5 days later and revealed worsening parameters despite empiric antibiotics.

Gram stain showed gram-positive bacilli and acid-fast stain was positive. CSF was analysed at the Indiana University microbiology laboratory via Mycobacteria Growth Indicator Tube (MGIT) liquid media while Nucleic Acid Amplification Testing (NAAT) was negative for *Mycobacterium tuberculosis *. Growth on solid media using Middlebrook 7H10 agar was observed and matrix-assisted laser desorption/ionization-time of flight (MALDI-TOF) MS identified the rapidly growing non-tuberculous *M. mucogenicum *. Parenteral therapy with amikacin 1 g every 24 h, cefoxitin 2 g every 6 h, and sulfamethoxazole-trimethoprim 15 mg kg^–1^ day^–1^ in three divided doses was initiated. The EVD was replaced and 4 weeks later a ventriculoperitoneal shunt was placed when repeated CSF studies were markedly improved (Table 1). The patient’s neurological exam improved and was nearly back to baseline 6 weeks later.

**Table 1.** CSF studies and cell counts

CSF parameters (normal range)	Day 5	Day 10	Day 28
Cell count (0–5 mm^–^3)	0 mm^–3^	80 mm^–3^	1 mm^–3^
Bands/neutrophils (0–2 %)	83 %	77 %	4 %
Lymphocytes (63–99 %)	4 %	3 %	20 %
Monocytes/macrophages (3–37 %)	13 %	20 %	76 %
Glucose (40–70 mg dl^−1^)	36 mg dl^−1^	28 mg dl^−1^	66 mg dl^−1^
Protein CSF (15–45 mg dl^−1)^	380 mg dl^−1^	452 mg dl^−1^	98 mg dl^−1^

## Discussion

*M. mucogenicum* is a gram-positive and acid-fast stain-positive NTM. It is classified as a rapidly growing mycobacterium (RGM) among the NTM due to its ability to grow within 7 days. In 1982, *M. mucogenicum* was first identified during an outbreak of peritonitis in two dialysis units and was called '* Mycobacterium chelonae *-like' organism [3]. In 1995, *M. mucogenicum* was designated a novel species based on 16S rRNA gene sequence analysis [8, 10].

*M. mucogenicum* is commonly isolated from municipal and hospital water supplies [6–8, 11]. It can form biofilms and replicate within protozoan hosts, and is resistant to standard disinfectants such as chlorine, formaldehyde, iodine and glutaraldehyde and to extreme temperatures [6, 9, 11–13]. The presence of this organism in tap water may contribute to the transient colonization or contamination of sputum samples but *M. mucogenicum* isolated in patients with neurological manifestation should not be considered a contaminant[14]. Catheter-related infections from *M. mucogenicum* are the most common healthcare-associated infections encountered. Contamination of the catheter during bathing was found to be the route of infection in several outbreaks [6, 11]. Our patient developed neurological and CSF biomarkers of meningitis within 1 week of placement of an EVD and responded to appropriate management.

*M. mucogenicum *, like other RGM, is resistant to first-line anti-tuberculosis agents (such as rifampin, rifabutin, ethambutol, isoniazid and pyrazinamide) but is sensitive to amikacin, cefoxitin, clarithromycin, imipenem and trimethoprim–sulfamethoxazole, ciprofloxacin, doxycycline and minocycline [15–17]. We opted for amikacin, cefoxitin and sulfamethoxazole–trimethoprim due to their more effective blood brain barrier penetration.

The usual treatment for *M. mucogenicum* is catheter removal combined with appropriate antibiotics for 6–12 weeks. An aminoglycoside combined with a macrolide and/or a fluoroquinolone is the most common empirical regimen. The duration of parenteral therapy is usually 2–4 weeks, followed by oral therapy for 4–6 weeks [2]. In our patient, despite improvement in clinical and CSF findings, we opted to continue antimicrobial therapy to the longer end of that range as there are no data on meningitis treatment for that pathogen.

## Conclusion

EVD-related infections of the CSF may have severe neurological sequelae and are difficult to treat. We report a unique case of *M. mucogenicum *-associated EVD-related meningitis.
